# Fanconi Anemia Patients Are More Susceptible to Infection with Tumor Virus SV40

**DOI:** 10.1371/journal.pone.0079683

**Published:** 2013-11-18

**Authors:** Manola Comar, Daniela De Rocco, Enrico Cappelli, Nunzia Zanotta, Roberta Bottega, Johanna Svahn, Piero Farruggia, Aldo Misuraca, Fabio Corsolini, Carlo Dufour, Anna Savoia

**Affiliations:** 1 Institute for Maternal and Child Health, IRCCS “Burlo Garofolo”, Trieste, Italy; 2 Department of Medical Sciences, University of Trieste, Trieste, Italy; 3 Haematology Unit, IRCCS Giannina Gaslini, Genova, Italy; 4 Pediatric Hematology and Oncology Unit, Oncology Department, A.R.N.A.S. Civico, Di Cristina and Benefratelli Hospitals, Palermo, Italy; 5 Pediatric Hematology Unit, Santobono Pausilipon Hospital, Napoli, Italy; International Centre for Genetic Engineering and Biotechnology, Italy

## Abstract

Fanconi anemia (FA) is a recessive DNA repair disease characterized by a high predisposition to developing neoplasms. DNA tumor polyomavirus simian virus 40 (SV40) transforms FA fibroblasts at high efficiency suggesting that FA patients could be highly susceptible to SV40 infection. To test this hypothesis, the large tumor (LT) antigen of SV40, BKV, JCV and Merkel Cell (MC) polyomaviruses were tested in blood samples from 89 FA patients and from 82 of their parents. Two control groups consisting of 47 no-FA patients affected by other genetic bone marrow failure diseases and 91 healthy subjects were also evaluated. Although JCV, BKV and MC were not found in any of the FA samples, the prevalence and viral load of SV40 were higher in FA patients (25%; mean viral load: 1.1×10^2^ copies/10^5^cells) as compared with healthy individuals (4.3%; mean viral load: 0.8×10^1^ copies/10^5^cells) and genetic controls (0%) (p<0.005). A marked age-dependent frequency of SV40 was found in FA with respect to healthy subjects suggesting that, although acquired early in life, the virus can widespread more easily in specific groups of population. From the analysis of family pedigrees, 60% of the parents of SV40-positive probands were positive for the virus compared to 2% of the parents of the SV40-negative probands (p<0.005). It is worthy of note that the relative frequency of SV40-positive relatives detected in this study was the highest ever reported, showing that asymptomatic FA carriers are also more susceptible to SV40. In conclusion, we favor the hypothesis that SV40 spread could be facilitated by individuals who are genetically more susceptible to infection, such as FA patients. The increased susceptibility to SV40 infection seems to be associated with a specific defect of the immune system which supports a potential interplay of SV40 with an underlying genetic alteration that increases the risk of malignancies.

## Introduction

Fanconi anemia (FA) syndrome, together with other genetic disorders and breast cancer 1 (BRCA1), a hereditary breast-ovarian cancer syndrome, are striking examples that the loss of genomic stability caused by a deficiency of the DNA repair pathways can confer an increased susceptibility to cancer [1]–[3].

FA is a rare autosomal or X-linked recessive disease with an estimated incidence of one to five per 1,000,000 live births and is caused by at least 15 genes [4]. These genes all cooperate in the DNA repair mechanism specialized in solving DNA interstrand crosslinks through a complex pathway that requires homologous recombination [1], [2].

The patients are at high risk of developing hematological and solid tumors at a young age. Specifically, FA subjects show an extraordinary risk for acute myeloid leukemia or myelodysplastic syndrome Non-hematologic tumors, including esophageal or vulvar and squamous cell carcinomas, mainly of the head and neck, are less frequent but have a higher distribution compared to the general population [5].

The polyomavirus simian virus 40 (SV40) is a DNA tumor virus that accidentally entered the human population through contaminated poliomyelitis vaccines during the period 1950–63 [6]. Contrasting reports have appeared in the literature on the presence of SV40 footprints in humans and its association with human tumors. Nevertheless, evidence linking SV40 to human cancer includes detection of SV40 DNA predominantly in brain and bone tumors, non-Hodgkin lymphoma and mesothelioma [7]–[9].

In cell transformation and tumorigenesis, the SV40 large antigen (LT) oncoprotein targets and inactivates the functions of key cellular products, such as the tumor suppressor p53 and the pRB family proteins. LT may also lead to transformation by inducing point mutations and chromosomal alterations, suggesting that the SV40 transforming properties depend on its ability to perturb tumor suppressor pathways, apoptotic processes and cell growth [10], [11].

It has been reported that SV40 transforms in vitro fibroblasts from FA patients more efficiently than normal fibroblasts [12] and additional recent observations have indicated that the SV40 LT induces overt DNA damage by activating the DNA damage response (DDR) [13]–[16]. Of note, the Fanconi anemia pathway, associated with replication stress, becomes activated by LT independently of the known Bub1 and pRB binding, since FancD2 accumulates in characteristic foci on chromatin. In addition, the stabilization of p53 by LT of SV40, presumably resulting from an activated DDR, can contribute to neoplastic transformation [17].

The mechanism by which tumor formation occurs in FA patients has not been determined although infections with Human Papillomavirus (HPV), a ubiquitous human tumor virus, has been recently considered an etiological event in these patients [18], [19]. To date, no population studies have been reported on the association between SV40 and FA disease.

The aim of this study was to investigate the prevalence of SV40 LT sequences in blood samples from a cohort of FA families, in order to evaluate a possible association of SV40 infection with FA syndrome. As a control, a comparative analysis was extended to human polyomavirus JC, BK and MC.

Our data show that a high number of FA patients and obligate carriers were positive to SV40 LT sequences. This finding seems to support the hypothesis that FA subjects showed higher susceptibility to SV40, raising a series of questions on the role of SV40 in the phenotypic outcome of FA patients. Moreover, it highlights the possible role of patients with a genetic dysfunction of the DNA repair mechanisms as source of SV40 infection in humans.

## Results

Genetic analysis using the DEB (depoxybutane) test [20] showed that, of the 89 probands, 71 belonged to complementation group FA-A, one to FA-B, two to FA-D2, and eight to FA-G. In the remaining seven probands, the mutated gene was unknown.

### Prevalence of Polyomavirus in FA Patients and Controls

Table 1 summarizes the prevalence data regarding SV40, JCV, BKV and MC LT detection in FA patients (No. 89), in no-FA patients (No. 47) and healthy individuals (No. 92).

**Table 1:** Prevalence of polyomavirus LT sequences in DNA samples from FA patients, no-FA patients and healthy controls.

		Polyomavirus			
Cohorts	No. ofindividuals	SV40	JCV	BKV	Merkel
		No. (%)	No. (%)	No. (%)	No. (%)
FA^a^	89	22^b^ (25%)*	0	0	0
no-FA^c^	47	0	0	0	0
Healthy	92	4 (4.3%)	1 (1%)	3 (3.3%)	0
Total	228	26 (11.4%)	1 (0.4%)	2 (0.9%)	0

a FA: Fanconi anemia patients.

b The DNA used for the analysis derived from different biological sources. The SV40 sequences were detected in DNA from 14 out of 54 (26%) peripheral blood cell samples, 8 out of 32 (16%) from lymphoblast cell lines and in none of 3 primary fibroblast cell lines.

* FA *vs* Healthy: *p: <0.005*.

c no-FA: patients with no-Fanconi genetic diseases: 17 Blackfan-Diamond anemia, 7 Shwachman-Diamond syndrome, 6 severe congenital neutropenia, 3congenital amegakaryocytic thrombocytopenia, 1 Pearson syndrome, 13 uncharacterized aplastic anemias.

The comparison of SV40 results, with the primers sets amplifying a Tag NH2-terminal coding sequence, revealed that the rate of SV40 carriers was 25% (22/89) in FA patients and only 4.3% (4/92) in healthy individuals [p<0.0005; O.R. = 5.7 (95% CI 2.0 to 15.8)].

JCV, BKV and MC LT sequences were not found in any of the samples from FA patients. BKV and JCV sequences were instead found in 3.3% (3/92) and in 1% (1/92) of healthy controls, respectively. Moreover, no polyomavirus DNA could be amplified in any sample from the no-FA group of patients.

The multiplex PCR analysis, conducted on the polyomavirus positive samples, detected by Q-PCR, confirmed the previous results. All controls without target DNA remained negative after each amplification run. In addition, 19/22 of LT SV40 positive samples (11 peripheral blood cells, 8 lymphoblast cell lines) from FA patients and 4/4 samples from healthy subjects were further analyzed with primers SVINTfor-SVINTrev allowing PCR amplification of intron sequences encompassing the Tag gene. Data showed that 15 out of 19 FA and 4 out of 4 healthy samples were also positive for the intron sequences.

Since the DNA samples from the FA patients were extracted from different biological sources, including cell lines, we could not exclude that exposure of the cell lines to the SV40 virus may have occurred during laboratory manipulations. For this reason, we evaluated SV40 positivity considering the origin of the DNA sample (Table 1). Specifically, we detected SV40 sequences in 14 out of 54 (26%) DNA samples purified from peripheral blood leukocytes, in 8 out of 32 (25%) samples from lymphoblast cell lines and in none of the 3 primary fibroblast cell lines, suggesting that SV40 detection was independent of the DNA source and that there had been no SV40 DNA contamination.

### Prevalence of SV40 in Obligate Carriers

**Figure 1:**
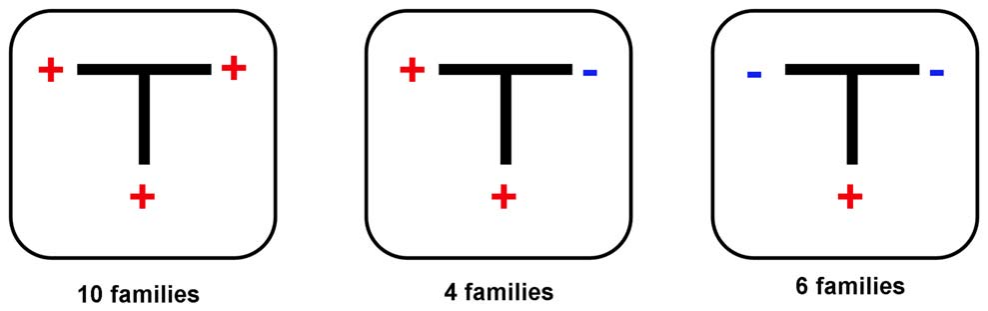
SV40 sequences in pedigrees of FA probands positive to SV40. DNA samples of parents were available for 20 families. In the trios, individuals positive to SV40 are indicated with “+” (red) and those negative with “−” (blue).

In order to test the susceptibility to SV40 in obligate carriers and evaluate the spread of the virus among family members, we tested DNA samples from peripheral blood cells (No. 68) and lymphoblasts cell lines (No. 14) of 82 parents of the FA probands. Twenty-five out of 82 (30.5%) subjects were found positive in the Tag NH2-terminal coding sequence (Table 2) and 24 of them showed also the Tag intron sequences. Interestingly, separating the obligate carries into two groups based on the “absence” or “presence” of SV40 in their offspring, we found that only 1 out of 42 (2%) parents of the SV40-negative patients had the virus (Figure 1). Among the parents of the SV40-positive patients, 24 out of 40 (60%) were positive to SV40 (SV40+parents *vs* SV40-parents: p<0.005). Of these 24 samples, half were from peripheral blood and the remaining half from lymphoblast cell lines. Analysis of the pedigrees of FA probands found positive for SV40 showed that the virus was present in both parents in 10 families, in one parent in 4 families (3 males and 1 female) and in neither parent in 6 families. For the remaining two probands the DNA samples of the family members were not available.

**Table 2:** SV40 infection in parents with respect to the presence of SV40 in their children.

FA patients	FA obligate carriers^a^	
	SV40+ No. (%)	SV40- No. (%)
**SV40+**	24/40 (60%)*	16/40 (40%)
**SV40-**	1/42 (2%)	41/42 (98%)
**Total**	25/82 (30.5%)	57/82 (69.5%)

a FA obligate carriers were from 44 families, both parents from 38 families and one parent from 6 families, accounting for 82 obligate carriers.

* SV40 positive obligate carriers in SV40+ parents *vs* SV40- parents: *p<0.005.*

### SV40 Viral Load in FA Patients

The quantification of the cellular single-copy of the beta-globin gene was used to normalize viral genome copies in DNA samples, corresponding to 100,000 cells. In the 22 SV40-positive patients, the virus DNA copies ranged from 1×10^1^ to 2.6×10^3^ copies/10^5^ cells. Specifically, in 12 samples the amount of viral load was <100 copies/10^5^ cells, in 3 from 100–1,000 copies/10^5^ cells and 7>1,000 copies/10^5^ cells. In the SV40-positive parents, the viral load was from 1×10^1^ to 3.3×10^3^ copies/10^5^ cells, while in the 4 positive healthy subjects it was less than 100 copies/10^5^ cells (Table 3).

**Table 3:** Distribution of SV40 LT viral load in FA probands and parents.

SV40 viral load copy number/10^5^ cells	FA probands No. (%)	FA parents No. (%)
**<100**	12/22 (54.5%)	7/25 (28%)
**100-1,000**	3/22 (13.6%)	5/25 (20%)
**>1,000**	7/22 (31.8%)	12/25 (48%)

### Correlation of SV40 Infection and FA Host Factors

In the attempt to define clinical or demographic factors in FA patients that could correlate with the presence of SV40, we considered the gene mutated in the FA probands. Of the 22 FA samples positive to SV40, 19 had mutations of FANCA and 1 of FANCG. The mutated gene is not known in the remaining 2 cases. The relative frequency of the positive SV40 samples was 27% and 13% for complementation groups FA-A and FA-G, respectively.

**Figure 2:**
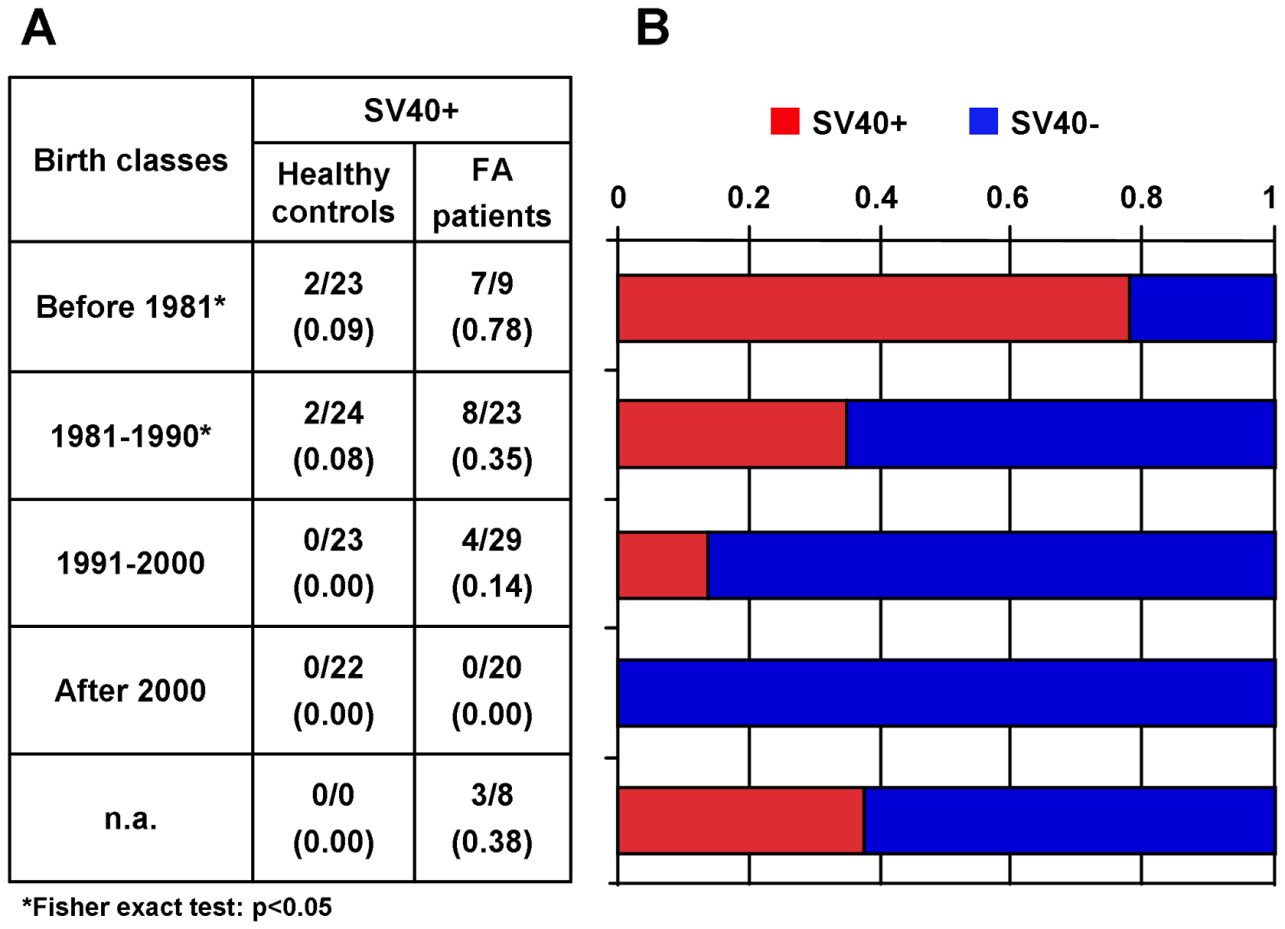
Birth class distribution of FA patients and healthy individuals. A) Table indicating the ratio of the SV40 positive individuals to the total of individuals tested. Healthy controls (No. 92) are matched with FA individuals (No. 84) by birth class. Years of birth for the class “before 1981” included 1 patient born in 1958 and 8 patients born between the years 1975 and 1980. n.a., year of birth not available. B) Histogram representing the relative frequency of the FA patients positive (red) and negative (blue) to SV40.

We also correlated the presence of the SV40 with the year of birth. Grouping the patients, as well as healthy controls, into 4 classes, we found that the percentage of patients positive to SV40 decreased linearly from the class of individuals born before 1981 to that of individuals born more recently (Figure 2). Of the 9 patients born before 1981, 7 out of 9 were positive to SV40. Among those born between 1981 and 1990 the number of positive cases dropped to 8 out of 23. For both classes the difference of positive cases between FA and healthy controls was statistically significant (p<0.05). A further decrease was observed in the group born in the next decade (1991–2000) with only 4 out of 29 positive cases. None of the 20 patients born after 2000 was positive to SV40. Even the 4 SV40-positive individuals from the healthy cohort belonged to the two oldest classes of age (Figure 2).

## Discussion

The analysis of blood samples from a cohort of FA patients for the presence of JCV, BKV, MC and SV40 LT antigen demonstrated that FA patients are particularly susceptible to SV40 infection. This represents the first in vivo description of a possible involvement of SV40 in this complex genetic disease. As reported by others for the Li Fraumeni syndrome [21] this data seems to support the potential interplay of SV40 with an underlying genetic alteration in the susceptibility to and development of malignancies.

In this study, SV40 LT sequences were detected in 25% of FA patients with a significant difference in the SV40 infection rate among matched controls groups (p<0.0005). To avoid the risk of false positive results due to SV40 plasmids contamination [9], another set of primers was employed. A slight difference in the results was found in samples with a low viral load (around 50 copies). This discrepancy was probably due to the different efficiency of the PCR primers employed to detect low level of the target sequences as shown by quantitative real time PCR.

This study suggests that SV40 is not merely an opportunistic virus in FA population. If it were, one would expect similar frequencies of detection in the control groups such as the no-FA patients, affected by other genetic bone marrow failure diseases not related to defects in DNA repair pathways, or the healthy subjects, where the peripheral blood mononuclear cells are semi-permissive for SV40 multiplication [22].

Based on these data, the following hypotheses arise. FA affected children are generally more susceptible to infections and have a decreased number of NK and B cells, with reduced cytotoxic function of NK and T cells [23]. Therefore, the increased susceptibility of these patients to SV40 infection could be associated with specific defects of the immune system activating the cellular DNA repair pathway which carry out SV40 replication [24].

The complex interplay of SV40 LT with the cellular DNA repair machinery involving the FA pathway could be responsible for the higher risk of tumor development observed in these patients. Consistent with our data, FA primary fibroblasts are 3- to 50-fold more sensitive to transformation by SV40 than normal fibroblasts [10], [23], [24]. Since effective cellular transformation requires integration of the virus, the increase in the transformation frequency suggests that the integration is more readily achieved in cells with a defective homologous recombination process [25]. SV40 infection has been shown to activate the ataxia telangiectasia mutated kinase, a master regulator of the DNA damage response that, acting as a barrier to cellular senescence and tumorigenesis, facilitates viral replication and persistence. It has been demonstrated that efficient productive infection requires RAD51 and, to a lesser extent, FANCD2, suggesting that homologous recombination is important for high-level extrachromosomal replication. Taken together, these data indicate that the SV40 LT antigen can be connected to and benefit from different players of the DNA repair processes [17].

Several studies have shown that SV40 is associated with human tumors, such as brain or bone cancers, non-Hodgkin lymphoma, leukemia and malignant mesothelioma [26]–[29]. Although cancer cases have been detected in our series of Fa patients during the follow-up (data not shown), the evaluation of SV40 in tumor tissues has not been possible. Nevertheless, evidence of tumor virus involvement in FA-related cancers was recently documented for HPV which prevalence in FA squamous cell carcinomas is greater than in the general population [18]. These data are consistent with the observation that the loss of the FA pathway stimulates both HPV oncogenic E7 protein accumulation and HPV genome amplification in differentiating cells, thereby demonstrating that the intact FA pathway functions to restrict the HPV life cycle [19].

Conversely, although 80% of the general population worldwide is seropositive for JCV, BKV and MC [30] we did not detect any specific sequences of these polyomaviruses in any of the FA samples tested. The absence of viremia in these patients is not an unexpected event. Although different technical factors such as distinct DNA isolation and PCR protocols, sample size, or pathogenetic status may influence prevalence data in the blood specimens, the recovery of PyV is basically linked to host’s immuno-survelliance and age. In this study we enrolled young FA patients (mean age 5.6 years) with a moderate immunodeficiency and for whom, a possible exposure to human polyomaviruses could not be still occurred [31].

We also found evidence of a high frequency of intrafamilial transmission of SV40. From the analysis of family pedigrees, only 2% of the parents of the SV40-negative probands were positive. The percentage grew up to 60% when the parents of SV40-positive patients were tested for the presence of the virus. It is worthy of note that the relative frequency of SV40-positive relatives detected in this study is the highest ever reported [32]–[34], suggesting that once in contact with the virus, even in asymptomatic FA carriers SV40 infection and persistence are frequent events. In this study, a marked age-dependent frequency of SV40 was found in FA cases, where a higher prevalence and viral load of the virus was found in older patients, born before 1981. In a recent serological study, we found a substantial number of SV40 seropositives in healthy people born between 1967 to 1978 [35]. It is less likely that SV40 antibodies in that group can be explained by vaccination, as individuals that age were at a relatively lower risk of exposure to contaminated poliovaccine, although there is evidence that, in some European areas, contaminated poliovaccines were used until the late 1970s [36]. Specifically, we also showed that SV40 VP antibodies in healthy children were at low prevalence (16%) suggesting that although acquired early in life, through different route, the virus is not widespread [37].

Data from this study seem to support a recently proposed model predicting contemporary SV40 infection in which SV40 infection is restricted geographically and demographically and influenced by sanitation and living conditions [38]. In addition to this, we favor the hypothesis that SV40 spread could be facilitated by individuals genetically more susceptible to infection, such as FA patients. In the case of FA defective DNA repair processes, the virus could infect cells more efficiently, persist longer and probably contribute to tumor induction in these patients. Future studies will be aimed at resolving the potential links between SV40 infection and the outcome of the FA phenotype also in different geographic areas.

## Materials and Methods

### Ethical Statement

Study approval was obtained from the Ethics Committee of the Gaslini Hospital, Genoa, Italy (protocol No. J5002 date: 24/9/2010). Written informed consent was obtained from all adults and from parents of the children involved in the study. All clinical investigations were conducted according to the principles of the Declaration of Helsinki.

### DNA Samples

Polyomavirus LT DNA was tested retrospectively on blood samples from 89 unrelated young Caucasian FA patients (mean age 5.6 years old) collected for molecular genetic evaluation between January 1992 and December 2011. The samples included DNA extracted from 54 peripheral blood cells, 32 lymphoblast cell lines and from 3 primary fibroblast cell lines.

DNA samples from peripheral blood cells (No. 68) and lymphoblast cell lines (No. 14) of 82 obligate carriers (both parents for 38 families and one parent for 6 families) were also analyzed.

As genetic control, DNA samples from 47 peripheral blood samples of no-FA patients affected with other bone marrow failure diseases, including Blackfan-Diamond anemia (No. 17), Shwachman-Diamond syndrome (No. 7), severe congenital neutropenia (No. 6), congenital amegakaryocytic thrombocytopenia (No. 3), Pearson syndrome (No. 1), and other uncharacterized aplastic anemias (No. 13) were included in the study. In addition, DNA from 92 peripheral blood cell samples of matched healthy volunteers (mean age 7.2 years old) were analyzed.

### Polyomavirus Sequence Detection

DNA samples were first assessed for suitability for PCR analysis by using a control reaction designed to amplify and quantify β-globin gene sequences [39]. DNA samples found positive for β-globin by PCR amplification were further investigated in duplicate for BKV, JCV and SV40 with primers and probes designed to detect sequences of 100 base pairs (bp) in the conserved N-terminal region of the Tag gene by quantitative real time PCR (Q-PCR), following the manufacturer’s instructions (RT Polyoma Panel kit, Eurospital Spa, Trieste, Italy). A new set of primers SVINTfor-SVINTrev, amplify a 235-bp intronic portion of the Tag gene were additionally used for SV40 detection. These primers are considered to be low risk for false-positive results due to putative contamination by laboratory plasmids containing SV40. The PCR cycle were 15 min at 94°C,, 45 s at 94°C, 45 s at 60°C, 1 min at 72°C for 40 cycles (9). PCR products were migrated in 1% agarose gel and visualized under UV light.

For MC detection, two different MC Tag regions, nt 571–879 and nt 1709–1846 were investigated by sPCR using the LT3F-LT3R and MCPyLT1709.F-MCPyLT1864.R primer sets, respectively. Amplicons of 308 bp and 138 bp are expected from these sPCR amplifications. Assay conditions for MC Tag sequence amplification were those reported by Goh et al. [40]. The assays can reproducibly detect 10 copies/reaction corresponding to less than 10 equivalents per reaction for the target sequence.

The positive samples were additionally confirmed with a multiplex type-specific polyomavirus PCR. Ten microliters of purified DNA was analyzed for the human PyVs BKV, JCV and MC, as well as the monkey PyVs SV40 by multiplex PCR using the Multiplex PCR Kit (QIAGEN), as previously described [41], [42]. Type-specific PCR primers target a conserved region of the large T-antigen gene at the N-terminal and amplify a DNA fragment of approximately 200 nucleotides. PCR products were obtained even when only 10 copies of the viral genome were used as template (data not shown). Typing of the specific polyomaviruses was carried out by hybridization of the PCR products to type-specific Luminex-bead coupled PyV probes, as described previously [43].

To confirm the reproducibility of the PCR assays and to avoid contamination, DNA was extracted with the recommended procedure for PCR investigation in a laboratory equipped with appropriate PCR facilities where the distinct phases of PCR were performed in separate rooms. Positive control plasmids were added to the control PCR reactions outside the core facility after tubes containing negative controls and test DNA were closed. Positive controls for polyomavirus PCR reactions were plasmid DNAs containing cloned SV40 (pSV40-B2E), JCV (pJC-MAD-1), BKV (pBKV-Dunlop) and MC (pMCPyVLT.1). Negative controls for the PCR assays were reactions without added DNA template.

### Statistical Analysis

Descriptive statistics were used to describe clinical and laboratory parameters. Univariate analysis was applied to assess the association between several possible explanatory variables, using the chi-square test or the Fisher exact test for categorical variables. Odds ratios (ORs) and 95% confidence intervals (95% CI) were estimated from the logistic regression coefficients and their standard errors.
